# Recommendations for cyberbullying prevention and intervention: A Western Canadian perspective from key stakeholders

**DOI:** 10.3389/fpsyg.2023.1067484

**Published:** 2023-03-07

**Authors:** Brittany P. Hendry, Laurie-ann Michelle Hellsten, Laureen J. McIntyre, Brenan R. R. Smith

**Affiliations:** ^1^Department of Educational Psychology and Special Education, University of Saskatchewan, Saskatoon, SK, Canada; ^2^Faculty of Education, The University of Winnipeg, Winnipeg, MB, Canada

**Keywords:** cyberbullying, prevention, intervention, stakeholder, education, restorative conferencing

## Abstract

**Introduction:**

Cyberbullying, or repeatedly communicating antagonistic messages using digital or electronic media meant to deal out harm or discomfort to others, has been considered more pervasive and impactful than traditional bullying since perpetrators can remain anonymous online, are not bound by time or place. In addition, cyberbullied youth are reluctant to involve others such as an adult or confront the perpetrator adults. Therefore, the primary purpose of this study was to capture a holistic understanding of potential youth cyberbullying prevention and intervention strategies (i.e., inhibiting forces that may reduce cyberbullying) from key stakeholders with professional knowledge about cyberbullying (i.e., educational administration, psychological counseling, technology and bullying education consultation, policing, research, and social support services).

**Methods:**

Twenty (*n* = 20) participants were recruited using purposive and snowball sampling techniques from both urban and rural school districts in one Western Canadian province to participate in either in a semi-structured individual interview (*n* = 16) or a scheduled focus group (*n* = 4) to achieve depth and understanding of cyberbullying issues. The I^3^ Model, a process-oriented metatheory of aggression with the potential to explain how cyberbullying behaviors continue to occur, was used as a frame to analyze the qualitatively gathered data using six phases of reflexive thematic analysis.

**Results:**

Participants identified educational efforts related to awareness of cyberbullying and consequences of perpetration, digital citizenship programming for students and social skills training, providing remediation to youth who are in online conflict with one another, and parental engagement with the technology used by their youth as key factors in mitigating instances of cyberbullying.

**Discussion:**

This study furthers research on cyberbullying prevention and intervention in schools by illuminating experiences from under researched and unique stakeholders in the field. These key findings and suggestions for future research are further discussed.

## Introduction

The explosion of mobile technology and ubiquitous access to the Internet has allowed for greater online connection and communication than ever before. Children as young as the age of two are now using internet-based communication technologies (Aslan, 2016). American statistics suggest that almost all United States teens aged 13–17 (95%) have access to a smartphone, almost half reported being online on a ‘near-constant basis’ (Anderson and Jiang, 2018), and 90% use social media (AACAP, 2018). However, a by-product of the proliferation of technological advancement and access is not without unintended consequences and anti-social behaviors can flourish such as bullying, harassment, and hate speech (Fulantelli et al., 2022). Electronic aggression can be characterized by the technologies and tools used to perform the actions (Nocentini et al., 2010), the identity of the victim (Pyżalski, 2012), or by relating the cyber aggressive behaviors to the paradigm of bullying (Cassidy et al., 2011). Cyberbullying, sometimes termed electronic bullying, e-bullying, mobile bullying, or digital bullying, is defined as “any behavior performed through electronic or digital media by individuals or groups that repeatedly communicates hostile or aggressive messages intended to inflict harm or discomfort on others” (Tokunaga, 2010, p. 278). Cyberbullying actions include derogatory messaging, threats, false rumors, photo modifications, masquerading, and exclusion (Broster and Brien, 2010). Cyberbullying has been considered more pervasive and impactful than traditional bullying, partly due to the ability for perpetrators to remain anonymous online and by the fact that cyberbullying is not bound by time or place (Patchin and Hinduja, 2006; Shariff and Hoff, 2007). Please review Waasdorp and Bradshaw (2015) for a comprehensive discussion on the overlap between traditional bullying and cyberbullying. Although global prevalence of cyberbullying has been difficult to determine, a recent global review suggests that the prevalence of cyberbullying has increased since 2015. Between 13 and 57% of children and youth have reported being a victim of cyberbullying while between 6 and 46% youth have engaged in cyberbullying perpetration (Zhu et al., 2021). Average global estimates of cybervictimization and perpetration are challenging to obtain due to wide variation in research methods, demographic characteristics, and differences in measurement. However, average global rates of perpetration are currently reported at approximately 25% and victimization at 33% (Zhu et al., 2021). Some of the highest rates of cybervictimization hover at 57% in Spain (Marco and Tormo-Irun, 2018), 52% in Malaysia (Marret and Choo, 2017), and 44% in China (Rao et al., 2019). Comparative to these higher estimates, lower rates of both perpetration and victimization have been observed in other parts of the world. In Canada and South Korea, victimization rates are estimated at 13 and 14%, while perpetration rates are estimated at 7 and 6%, respectively (Beran et al., 2015; Lee and Shin, 2017). The alarming state of cyberbullying prevalence among adolescents is compounded by the fact that more than half of cyberbullied youth report they do nothing in response to their victimization (i.e., do not tell a trusted adult or confront the perpetrator; Mishna et al., 2010). This reluctance by youth to involve adults to aid in problems of cyberbullying is especially important, as there are many health-related consequences of prolonged cyberbullying victimization.

### Consequences of cybervictimization for young people

There is an abundance of literature demonstrating negative outcomes for youth related to cyberbullying. A review of the literature has revealed that cyberbullying can be detrimental to the health of adolescents and is considered an emerging public health concern (Nixon, 2014). The compromised health conditions tied to cyberbullying are related to the emotional, social, behavioral, and even physical domains of a youth’s life. As a result of cybervictimization, youth may experience numerous emotional challenges including: increased anger and sadness (Beran and Li, 2005; Patchin and Hinduja, 2006), depression (Campbell et al., 2012; Bonanno and Hymel, 2013; Chang et al., 2013), and anxiety (Wigderson and Lynch, 2013). Youth also tend to experience negative social consequences from victimization, such as increased social anxiety (Juvonen and Gross, 2008; Dempsey et al., 2009), increased loneliness (Devine and Lloyd, 2012; Olenik-Shemesh et al., 2012) as well as problems with peers and having fewer friendships overall (Price and Dalgleish, 2010; Jackson and Cohen, 2012). In addition, youth may experience behavioral changes as a result of cybervictimization. Research demonstrates that young people who are perpetually victimized in the cyber world are at risk for increased violent behaviors at school, delinquency, and substance use (Hinduja and Patchin, 2007, 2008; Ybarra et al., 2007; Goebert et al., 2011). Unfortunately, cybervictimization has also been shown to increase suicidal ideation and suicidal behaviors (Hinduja and Patchin, 2010; Bonanno and Hymel, 2013; Litwiller and Brausch, 2013). With the potential for such grave consequences of cybervictimization, educators and healthcare professionals should be aware of prevention and intervention efforts that may reduce cyberbullying behaviors among youth.

### Efforts to prevent cyberbullying and cybervictimization

Several isolated components of anti-bullying and/or anti-cyberbullying programs have demonstrated the ability to reduce rates of bullying and victimization by approximately 20% (Ttofi and Farrington, 2011). Research has shown that some program components and protective factors seem to be the most influential in reducing bullying and victimization. Parental engagement and parenting strategies have consistently demonstrated an important role in the reduction of bullying and victimization. For example, in a review of parental roles and cyberbullying among youth, Elsaesser et al. (2017) found certain mediation strategies for controlling Internet and technology use were more effective than merely placing blanket restrictions on youth. When youth are involved in creating the rules about Internet and technology use, rates of cyberbullying and cybervictimization tend to decrease. In contrast, parents who are more controlling and restrictive about Internet and technology use only lead to minimal reductions in cyberbullying and cybervictimization. These findings may highlight the issue of ideal parenting approaches in relation to youth cyberbullying, as parents who exert high warmth and control (i.e., authoritative parenting) are associated with lower rates of cyberbullying perpetration compared to parents who exert low warmth and high control (i.e., authoritarian parenting; Elsaesser et al., 2017). Additionally, program intervention strategies that target parents are some of the most effective approaches to combat bullying (Ttofi and Farrington, 2011; Roberto et al., 2017) and scholars have recommended it is important to continue targeting parents in order to reduce bullying (Hutson et al., 2018).

Other intervention strategies used to reduce bullying and cyberbullying have been investigated. Project-based learning strategies to raise awareness of cyberbullying have shown positive outcomes, such as increased vocabulary, knowledge, and awareness of the consequences of online behaviors (Chen, 2018). Additionally, anti-cyberbullying messaging and policy/practices that help persuade young people to safely use the Internet and seek social support for cyberbullying issues have shown reductions in cyberbullying rates and susceptibility to cyberbullying (Ortega-Ruiz et al., 2012; Savage et al., 2017). Other strategies to reduce cyberbullying behaviors include school-based approaches focused on traditional bullying. Although traditional bullying and cyberbullying may be defined differently (Selkie et al., 2016), some researchers have found that general bullying prevention programs have been effective in reducing cyberbullying and cybervictimization as well (Gradinger et al., 2015). For example, based on a meta-analysis of anti-bullying programming, Ttofi and Farrington (2011) found incorporating disciplinary methods (e.g., deprivation of special privileges, stern discussions with bullies), teacher training, classroom management, and cooperative group work was effective in reducing traditional bullying perpetration and victimization. Some of these methods outlined by Ttofi and Farrington (2011), such as disciplinary action and teacher training, could be applied to a school-based cyberbullying prevention/intervention strategy, but have yet to be comprehensively investigated.

Although prevention and intervention efforts that aim to reduce traditional bullying have been on the rise throughout the last decade, more evidence is needed to ascertain if those same principles can be applied to cyberbullying prevention and intervention. While there is considerable overlap between traditional bullying and cyberbullying, some research suggests that negative outcomes of cybervictimization have been significant even while controlling for involvement in traditional victimization (Perren et al., 2010). This indicates a need for cyberbullying-specific prevention and/or intervention efforts, but scholars note that these efforts have not been well-researched to date (Tanrikulu, 2018). Two recent systematic reviews have investigated the components of intervention programs and methods for cyberbullying specifically (Hutson et al., 2018; Tanrikulu, 2018). According to Hutson et al. (2018), the most commonly implemented program components include: improving digital citizenship, collaboration, communication and social skills, empathy training, education on cyberbullying, enhancing coping skills, and peer mentoring. However, Tanrikulu (2018) found that the program duration, instruments to measure cyberbullying, and theoretical program bases varied widely with no clear pattern of common program components. Such wide variation among programming makes it difficult to compare and determine which programs are most effective. While some approaches for cyberbullying prevention and intervention currently exist, research in cyberbullying prevention and intervention is inconsistent in terms of implementation and evidence.

### The current study

As Ioannou et al. (2018) indicated, cyberbullying research is dominated by self-reported measurement, which can enhance issues of social desirability, personal interpretation, and a divergence between reported behavior and actual behavior (Coughlan et al., 2009). Qualitative research in this domain would allow for a more holistic understanding of the experience and perception of key stakeholders involved in the cyberbullying world (Tracy, 2013). While purely qualitative methods in cyberbullying research are increasing, many studies focus on the youth perspective of perceptions and the experiences of cyberbullying itself (e.g., Vandebosch and Van Cleemput, 2008; Evans et al., 2016; Ghazali et al., 2017; Chia-Wen et al., 2019). Critical key informants and stakeholders who have experience managing cyberbullying issues on a regular basis may be the key to adequately addressing, designing, and implementing prevention strategies to reduce cyberbullying. Ioannou et al. (2018) offered practical suggestions for future work in cyberbullying research that highlighted the currently non-existent collaboration and dialog between multiple communities with stake in the cyberbullying world. These groups may include experts from computer science, psychology, and sociology to better shed light on the complex issue of cyberbullying. Additionally, much of the current research that qualitatively consults individuals that are not adolescents include mainly parents, school administrators, and teachers (e.g., Noah, 2012; Ragain, 2014; Young et al., 2017). The potential for unique and vital perspectives to exist outside of the view of parents, teachers, and youth warrants more exploration (Pennell et al., 2020). Furthermore, investigating this issue through the lens of multiple experiences allows for a more holistic understanding, as Couvillon and Ilieva (2011) emphasized: “cyberbullying intervention requires the joint efforts of everyone who shares concerns about the safety and children of youth” (p. 98).

As much of the cyberbullying research has been conducted in the absence of theory (Tokunaga, 2010), our research was guided by the theoretical framework of the I^3^ Model (Finkel, 2014). The I^3^ Model is a process-oriented metatheory of aggression that has the potential to explain how cyberbullying behaviors continue to occur and has been successfully applied in recent cyberbullying queries (Wong et al., 2018). This framework is useful in cyberbullying research as it illuminates how non-aggressive interactions may become aggressive based on three interrelated processes: inhibiting forces, impelling forces, and instigating triggers. *Inhibiting forces* are factors that decrease the likelihood of an aggressive response (e.g., ability to exercise adequate self-control in response to aggression)*. Impelling forces* are influences that determine the overall strength of the response (e.g., the belief that the perpetrator is truly anonymous). Finally, *instigating triggers* are the situations that increase the likelihood of an aggressive response (e.g., experience as prior victim of cyberbullying). The I^3^ Model posits that if instigation and impellence are heightened and inhibition is decreased, aggressive responses will surface. Therefore, in terms of prevention and intervention, it is pertinent to understand what potential inhibiting forces are recommended to reduce cyberbullying among youth.

The primary purpose of this research was to capture a holistic understanding of potential youth cyberbullying prevention and intervention strategies that are suggested by key stakeholders. To achieve this, we aimed to incorporate multiple unique, but vital, voices within the cyberbullying world that have yet to be demonstrated in formal research. Key voices need to be stakeholders who have both direct youth connections (school administrators, guidance counselors, consultants, student support professionals), and indirect youth connections (school resource police officers, bullying educators, and cyberbullying researchers). Through the lens of the I^3^ Model, this study examined stakeholder suggestions that serve as inhibiting forces and may reduce cyberbullying. Potential barriers to the prevention and intervention of cyberbullying issues are also explored.

## Materials and methods

### Participants and procedures

To achieve depth and understanding of cyberbullying issues, qualitative data were collected from one-on-one semi-structured interviews (*n* = 16), as well as one focus group (*n* = 4). Participants were recruited from both urban and rural school districts in one Western Canadian province. Purposive and snowball sampling techniques were employed to target key stakeholders with professional knowledge about cyberbullying. Targeting key stakeholders with professional knowledge allowed for their experiences to be deconstructed and interpreted for a better understanding of this complex phenomenon (Tracy, 2013). A trained graduate student with extensive experience in qualitative interviewing collected all data. The focus group and interviews included key stakeholders representing professions related to educational administration, psychological counseling, technology and bullying education consultation, policing, research, and social support services. Table 1 outlines the participants and occupation descriptors. Participant recruitment ceased when the research group observed redundant responses and perspectives, which indicated data saturation (Mills and Gay, 2016). Interviews were approximately 60 min in length and interview questions related to cyberbullying methods and motivations (e.g., what are the technological means through which adolescents are cyberbullying each other?), victim and perpetrator characteristics (e.g., are there particular reasons why certain adolescents are more likely to cyberbully and/or be cyberbullied?), how stakeholders currently viewed cyberbullying (e.g., how is cyberbullying similar or dissimilar to traditional bullying?), recommended prevention and intervention strategies to successfully mitigate cyberbullying issues (e.g., are you aware of alternate measures through which cyberbullying is being successfully addressed?), and any factors (e.g., institutional, legal) that stakeholders believed hindered the prevention and/or intervention of cyberbullying (e.g., are the school and justice systems equipped to properly prevent and/or intervene in instances of cyberbullying?). These questions were presented in a general way in order to allow for participants to deviate from the interview schedule and illuminate their experiences as they presented (Lee, 1999).

**Table 1:** Participant and occupation characteristics.

Participant	Occupation title(s)	Occupation description(s)
Focus group (*n* = 4)	Principal (1), school counselor (1), youth social support workers (2)	Professionals who provide oversight and services related to educational policy, curriculum implementation, administration, and counseling and support services to students.
1	Superintendent of Education	An educational professional who has oversight into the implementation of policy, curriculum, and management of facilities. Primary liaison between the provincial government and school districts.
2	Principal	An educational professional in charge of administration of the entire school (grades K-8), disciplinary actions, resource management.
3	Principal	An educational professional in charge of administration of the entire school (grades 9–12), disciplinary actions, resource management.
4	Vice Principal	An educational professional in charge of daily administrative elements of the school (grades 9–12). Oversight of scheduling, registration, and disciplinary actions.
5	School counselor	A mental health professional that provides direct psychological counseling to students (grades 9–12), make referrals to community programs, address student needs.
6	School counselor	A mental health professional that provides direct psychological counseling to students (K-12), provide skills workshops to students, mental health education, facilitate anxiety, and depression groups.
7	School counselor	A mental health professional that provides direct psychological counseling to students (K-8), resolve social tensions between students, provide skills workshops/presentations.
8	Instructional technology consultant	A professional who works for the Ministry of Education and provides professional development programs for teaching staff related to technology.
9	Bullying educational consultant	An educator within the private sector that provides programming to students about peer respect and bullying prevention.
10	Bullying researcher	A researcher in sociology that examines youth delinquency and bullying/cyberbullying.
11	Police officer	A police officer in the school resource unit; primary liaison between staff, students, and parents in high schools (grades 9–12) and elementary schools (grades K-8).
12	Police officer	A police officer in the school resource unit; conducts risk assessments, conducts home visits, facilitates police resources between all schools in the district.
13	Police officer	A retired police officer from the school resource unit; provided liaison between staff, students, and parents in high schools (grades 9–12) and elementary schools (grades K-8).
14	Student support professional	A social work professional who works closely with schools (grades 9–12) to support students in areas of conflict resolution, bullying, relationships, and facilitates mediation between students.
15	Student support professional	An educational professional who works closely with schools (grades 9–12) to support students in areas of conflict resolution, bullying, relationships, and facilitates mediation between students.
16	Student support professional	A social work professional who works closely with schools (grades 9–12) to support students in areas of conflict resolution, bullying, relationships, and facilitates mediation between students.
*N* = 20		

### Data analysis

The data were imported and analyzed in NVivo 12 Pro. Upon verbatim transcription of the interviews, the data were reflexively thematically analyzed, where larger themes are subsequently broken down and refined into sub-themes that represent the message of the participants using six steps or phases: familiarizing self with data; coding; generating initial themes; developing/reviewing themes; refining, defining, naming themes; and writing it up (Braun and Clarke, 2006, 2022). Multiple researchers conducted the analysis independently before jointly agreeing on the resulting themes. Regular meetings to discuss and refine emerging themes took place several times over the course of the analysis phase.

### Findings

Several inhibiting forces were identified which were believed to decrease the likelihood of online aggression related to cyberbullying issues. First, participants suggested that educational efforts related to awareness of cyberbullying and consequences of perpetration were paramount. Digital citizenship programming for students and social skills (empathy, respect, conflict-management) training were also fundamental to decreasing online aggression. Additionally, providing remediation to youth who are in online conflict with one another was suggested as a highly effective form of intervention. Last, participants emphasized parental engagement with the technology used by their youth was also key in mitigating instances of cyberbullying.

### Education

All participants indicated that educational strategies are the foundation to decreasing cyberbullying issues among youth. The educational strategies include providing students’ awareness about cyberbullying and the potential legal consequences of cyberbullying perpetration, digital citizenship programming for youth, as well as social skills training in empathy, respect, and conflict management.

#### Ongoing education and awareness of cyberbullying

The majority of participants described the importance of an ongoing education program and awareness of cyberbullying as an effective means of prevention. One school counselor emphasized the importance of ongoing anti-bullying education, “I think we have to continue to educate kids and cannot just stop at Grade 9—we cannot assume that because we have done this presentation once that we do not have to keep doing it every once and awhile.” Another school counselor echoed similar sentiments about the impact of education on cyberbullying and how educational programming has increased awareness and communication about cyberbullying. The school counselor stated, “The fact that we have some anti-bullying thing and the pink shirt day, now we do the bullying awareness…I think those have been very helpful to bring it out into the open where people are actually talking about it.” While spreading awareness of cyberbullying was emphasized, the awareness of legal consequences to cyberbullying perpetration was also recommended.

#### Awareness of legal consequences

Another suggested educative lesson was the understanding that cyberbullying behaviors have the potential to become legal or criminal issues. One school counselor described how they have implemented an informational presentation for students about the potential legal consequences of cyberbullying stating; “It’s always as an educational component where the police are saying to the student or the youth in their family that if this continues, this is where this could end up. This is what you can be charged with.” Additionally, one police officer involved in providing legal education to students believed it was important to convey an awareness of the consequences of cyberbullying asserting; “One of the messages I try to get across is consequences. Negative consequences for negative actions and treat others the way you want to be treated.” In addition to informing youth of the potential legal ramifications for cyberbullying behaviors, structured programming to foster good digital citizenship was also recommended.

#### Digital citizenship programming

All participants indicated the importance of offering general digital citizenship programming as a method to prevent negative behaviors online. The aim of this programming is to ensure that students know how to act as good digital citizens. One police officer outlined, “…Instead of burying our heads, we should be teaching kids how to use these things…basically what we are talking about is being good citizens; it’s not even digital citizenship, it’s citizenship and we are teaching them but how online it should be.” When asked about the value of teaching youth digital citizenship, a school technology consultant also stressed the importance of modeling digital citizenship and emphasizing positive interactions online:

It’s so important for us to actually take kids to online spaces and to model how we interact in those online spaces… that’s how we learn behaviour…if we don’t take kids to those online spaces and give them the opportunity to see us model and interact in those online spaces, then they don’t have that benchmark.

While having youth understand the importance of being a good digital citizen was noted as being a key to reducing cyberbullying, providing social skills training was also considered imperative.

#### Social skills training

The majority of participants believed that educating students in certain social skills would reduce cyberbullying events. These social skills include teaching youth about empathy, respect, and conflict management. When describing the significance of having students respect each other in online spaces, one guidance counselor noted: “…so I think that it all starts with that right? Treating other people with respect.” One police officer who was responsible for providing cyberbullying education to students and teachers highlighted that learning how to empathize with others should be at the forefront of cyberbullying prevention:

My big message now is empathy. You have no idea who the other person is sitting beside you. Empathy is massive…if you empathize, you walk in their shoes, you feel their pain, you make change. One person can change the world and that’s the message I’m pounding out now.

A school technology consultant reiterated that in addition to learning how to empathize with others, learning to respect others as a skill is important:

We come from that pro skill-based approach where it’s like, ‘let’s show them what they need to do.’ We can’t just tell people, ‘don’t be a bully, don’t be a bully.” If we’re gonna say that, we need to replace that with what behaviour we need…we want people to understand respect… at the end of the day I think we come down to teaching those skills we want for people.

In addition to teaching respect and empathy, participants also noted that teaching conflict management is crucial. A police officer who worked within schools noted that we need to “teach kids skills on how to deal with conflict because it is a part of life.” Similarly, an instructional consultant for anti-bullying messaging described a similar need to teach youth the skills to handle conflict:

We want them to understand that conflict is natural. We don’t want them to stop having conflict. That’s just going to be part of being human. But how that conflict is resolved, and how that conflict is resolved when there is a power differential, is important.

Efforts toward educating youth on cyberbullying awareness, being a good digital citizen, and providing social skills training were noted as being mostly preventative. However, key stakeholders noted that remediation has been highly effective in intervening when instances of cyberbullying come to fruition.

### Restorative conferencing

The majority of participants mentioned efforts to provide students restorative conferencing was an effective intervention/response to cyberbullying incidents. School counselors, student support professionals, and police officers were the most frequently involved in remediation between youth. Our participants described remediation for cyberbullying incidents as gathering all involved in the conflict, discussing the situation, and creating a peaceful plan to end the conflict. One police officer described why bringing students together during conflict is essential:

If there is a dispute–one is bullying the other…it gives them a chance to explain why they were doing it, but also the victim too. You know, see how it made them feel and what have you done, and hold them to more account. I think it is pretty effective.

A student support professional also endorsed this strategy when discussing the most effective ways to intervene in cyberbullying issues: “A lot of the online stuff goes away when they [students] have a chance to be able to communicate and listen to the other person and have a chance to respond.” Similarly, a vice principal who did not have access to professional support for restorative conferencing described a similar process that their school implements in response to cyberbullying problems:

We would want to bring the other party in a non-confrontational way, make the person aware of the effect of those messages…the student who has sent the message becomes aware or is made aware that the message that they’ve sent is injurious, and whether they know it ahead of time or not…they come to understand the full effect of that kind of a message.

When cyberbullying instances occur, it was clear that our participants felt that the most effective form of intervention was mediating the conflict between students. However, another effective strategy to reduce cyberbullying involve parental engagement with technology used by their youth.

### Parental involvement with technology

All participants identified parents as a significant resource to reduce and/or resolve cyberbullying conflicts between youth. Our participants were most likely to discuss parental involvement with technology, specifically, as a strategy to prevent or intervene in situations of cyberbullying. One student support professional described a common situation where parents may overestimate the maturity and responsibility of youth in relation to their technology use:

Some parents are like ‘woo they made it to high school, I’m out of here, bye. Suppers at 6:00,’ kind of feel… ‘thank god they made it to high school, and now they’re old enough and mature enough, they don’t need me anymore’.

This student support professional conveyed that while parents have the best intentions, this inadvertent release of control tends to escalate and mismanage issues of cyberbullying. When asked about a recommended strategy for reducing cyberbullying, one police officer suggested, “Parental controls, parents taking more responsibility in their usage of their children’s usage online, them monitoring what’s being said, pictures being posted and shared, parents need to take a bigger role instead of just trusting their kids.” A similar sentiment was echoed by a different student support professional, when they described the importance of parents taking an active role of responsibility in their child’s technology use, “I think it’s important for parents to set boundaries. At a young age. In terms of having smartphones, in terms of having access to social media.” Parental involvement in youth’s use of technology was also discussed as a punitive strategy upon intervention. As one school counselor described” “Some of the parents then put some restrictions [on the offenders]: some lost their phones, their accounts, blah blah blah. So the parents took a more active role in what their kids were using their technology for.”

## Discussion

Cyberbullying remains a significant concern for the physical and emotional health of youth, yet it continues to infiltrate our communities. Thus, it remains imperative to pinpoint effective strategies to curtail the negative effects of cyberbullying. The purpose of this study was to outline the prevention and intervention strategies for cyberbullying suggested by key stakeholders through the theoretical lens of the I^3^ Model. The results of this study illuminated key inhibiting forces that theoretically should decrease the likelihood of aggressive online responses, such as those involved with acts cyberbullying.

As previously noted, cyberbullying remains a relatively new phenomenon. As such, there has been limited investigation of different cyberbullying-specific prevention/intervention efforts in the academic literature to date. Nonetheless, the results of our research still complement other key findings in the literature. Our participants emphasized that ongoing education and awareness was vital to prevent instances of cyberbullying. Other research on cyberbullying prevention programs and strategies echoed similar recommendations, where prevention work needs to be routine and ongoing (Mason, 2008; Couvillon and Ilieva, 2011). Fortunately, other stakeholders in the literature appear ready to embrace such educational endeavors in their schools. In a study examining the opinions of teachers and parents about cyberbullying prevention, Gradinger et al. (2017) found that 95% of parents and 90% of teachers have positive opinions regarding facilitating and participating in anti-bully education strategies. Consequently, it is likely that teachers and parents would be supportive and invested in implementing such educative programming. Other research has already documented that teachers and educational support professionals have indicated a desire to receive additional training related to cyberbullying interventions (Bradshaw et al., 2013). Additionally, Yanagida et al. (2019) investigated the effectiveness of an anti-bullying program and acknowledged the value of implementing such education. However, Yanagida et al. (2019) noted that existing anti-bullying programs may need to be modified to address features of cyberbullying. The results of this study can speak to those suggestions, as cyberbullying awareness, digital citizenship training, and empathy, respect, and conflict management exercises can be easily implemented as new modules within existing anti-bullying programs. Lastly, similar to other researchers (Tanrikulu, 2018), ideas from key stakeholders in this study suggest educational efforts and anti-cyberbullying/bullying programming within schools is likely the best option for diminishing cyberbullying.

Traditional efforts to end conflict within schools, such as zero-tolerance bullying policies, continue to be practiced despite theories suggesting these policies contribute to the ‘school to prison pipeline’ phenomenon, where using punitive measures in schools push students toward the criminal justice system (Hirschfield, 2008; Berlowitz et al., 2017). Experts have argued that such policies are ineffective and lead to negative impacts in Canadian schools (Daniel and Bondy, 2008). A significant finding in this study is that key stakeholders felt that remediation efforts (e.g., restorative conferencing) were highly effective in managing cases of cyberbullying and identified restorative conferencing as an effective cyberbullying intervention. To date, the literature is limited in regard to how specific restorative justice tools have been applied to resolve school conflicts including cyberbullying (Morrison et al., 2005). However, Duncan (2016) and Das et al. (2019) both propose using features of restorative justice principles as a practical solution for schools with Duncan suggesting that ‘restorative practices hold great promises for many cases’ (p. 254). Das and colleagues recommend the creation and use of a virtual peace room (similar to an online chat room), and a restorative justice coordinator to facilitate interactions between conflicting parties. Duncan (2016) advocates for family group conferencing, led by a trained facilitator, when all individuals willingly participate. However, Duncan (2016) conceded that challenges to the implementation of restorative justice programs include related time requirements and financial cost, which may be partially offset by donors or sponsors. Three participants in this study were non-teaching stuff employed by a non-governmental organization through sponsored funds to provide social support to students and families. These individuals are situated directly within the school (one facilitator per school or shared among two schools). Part of their professional role is to provide youth and families conflict resolution strategies and facilitate restorative conferences between affected parties when social issues (e.g., [cyber]bullying, home conflict) emerge in the school. The restorative conferences carried out by these participants are best defined as a structured, victim-sensitive meeting involving all victims, offenders, and family/friends to address a wrongdoing and problem solve together to repair harm done (O’Connell et al., 1999). Similar to Duncan (2016), the results of this study suggest that despite the potential added costs to successfully intervene in instances of cyberbullying among students, schools should consider employing a restorative justice approach.

Another significant aspect highlighted in this study is the importance of parental involvement with their youth’s technology usage. Our key stakeholders emphasized that parents tend to overestimate their child’s maturity, level of responsibility, and boundaries with technology. Other research has shown that youth are less likely to participate in bullying behaviors when they have parents and teachers who clearly outline that those behaviors are not appropriate (Hinduja and Patchin, 2013). The outcry for parents to become more involved in their children’s technological lives is not just emphasized by our participants. Cassidy et al. (2018) found that educators held strong beliefs that parents lacked awareness of their youth’s online behaviors and activities, and generally failed to monitor their youth’s online presence. Other researchers have documented that parents who have low levels of knowledge about their children’s whereabouts and activities are associated with higher delinquent behaviors by the youth (Laird et al., 2003). These findings suggest that parents may be able to help reduce cyberbullying by providing more oversight and through involvement in their youth’s [technological] lives. However, the types of oversight and rules imposed by parents should be chosen carefully. Elsaesser et al. (2017) found that parents who created rules with their youth around technology use, as opposed to merely restricting access to technology, were more effective in reducing rates of cyberbullying. While this research recommends parents become more involved in their youth’s online lives, pragmatically doing so may be difficult as parents have noted that they do not wish to infringe in their children’s privacy or lack the skills to supervise such online activities (Monks et al., 2016).

### Practical implications

Although a comprehensive review by Tanrikulu (2018) was unable to discern a consistent pattern of cyberbullying prevention/intervention programs or components, the recommendations by key voices in this study were consistent. Education (awareness, digital citizenship training, and social skills training), restorative conferencing, and parental involvement with technology are fundamental pillars to reducing cyberbullying behaviors. Our research and others echo stakeholder emphasis of prevention through education. Broll and Huey (2015) interviewed 12 police officers (school resource officers and patrol officers) in Southwestern Ontario about their perspectives on cyberbullying issues. Similar to our research findings, the police officers’ emphasized education about safe and appropriate technology use and parental involvement/monitoring of technology are necessary to prevent cyberbullying. Additionally, when deliberating best practices to address cyberbullying issues among youth, it is uncommon to hear from the voices of outside or indirect stakeholders, such as the non-teaching student support professionals and police officers that are included in this study. Consulting these unique collaborators may continue to offer novel solutions to cyberbullying concerns. The implications of this research have the capacity to inform strategic programming efforts to include lessons on cyberbullying awareness, digital citizenship, knowledge of legal consequences for cyberbullying perpetration, and social skills training. This research can also better inform funding decisions for school districts. If restorative conferencing is the most effective tool to address cyberbullying issues between students, financial decisions to include a school support worker to offer these services may be vital. Last, this research illuminates important information for parents of youth who have access to technology. A recent review highlighted that less than half of the developed anti-cyberbullying programs incorporate educational content for parents, although such programs are among the most successful at reducing cyberbullying and cybervictimization (Hutson et al., 2018). In places where anti-cyberbullying education is unavailable to parents, our research suggests that merely empowering parents to take an active role in their youth’s online presence could potentially reduce and/or prevent cyberbullying incidents from occurring.

### Limitations and future research

Although the results of this study provided suggestions and important implications, this study is not without limitations. This study brings a Canadian perspective to a research area that is predominantly American and European; however, the findings of this study are based on a sample from a limited geographical area (one province). Therefore, samples of key stakeholders in other parts of the country and/or world should be investigated before adopting these recommendations into practice. Additionally, the sample size is limited and therefore not completely representative of all key stakeholders who work with youth and schools. One future direction could be to use the results of this study to create a survey to confirm these findings with a broader sample. In addition, one of our main findings was that providing restorative conferencing for youth experiencing conflict can help reduce and/or resolve cyberbullying in schools. However, only some of the stakeholders in our sample had access to this type of a resource. Because restorative conferencing can occupy a great deal of time and financial resources and is typically supported by non-educative school personnel, not all schools will have the personnel, expertise, or funding to facilitate these types of conferences. Future research should continue to ask unique stakeholders (e.g., police officers, student and family support professionals) about other types of potential school supports available in their particular geographic area as the responses may provide different suggestions for solution. Last, while much of the cyberbullying literature remains atheoretical, this study is one of few to employ the I3 Model to target the inhibiting forces that reduce cyberbullying, specifically. Therefore, it is recommended that future research continue to apply the I3 Model to assess the overall utility of its use within cyberbullying research.

## Conclusion

This study sought to capture a holistic understanding of potential youth cyberbullying prevention and intervention strategies (i.e., inhibiting forces that may reduce cyberbullying) from key stakeholders with professional knowledge about cyberbullying (i.e., educational administration, psychological counseling, technology and bullying education consultation, policing, research, and social support services) through the theoretical lens of the I^3^ Model. The perspectives and opinions of partners with indirect connections to youth are rarely sought, which contributes to the uniqueness of the study. Furthermore, these novel perspectives suggest promising approaches (e.g., restorative conferencing) to successfully intervene in cyberbullying incidents. Participants identified educational efforts related to awareness of cyberbullying and consequences of perpetration, digital citizenship programming for students and social skills training, providing remediation to youth who are in online conflict with one another, and parental engagement with the technology used by their youth as key factors in mitigating instances of cyberbullying. These findings illuminated key inhibiting forces that theoretically should decrease the likelihood of aggressive online responses, such as those involved with acts cyberbullying. The reduction and/or prevention of cyberbullying incidents from occurring should continue to be a focus of current research to better understand the challenges youth face and the supports they need to function in today’s technology focused world.

## Data availability statement

The datasets presented in this article are not readily available because this project remains in progress and data analysis is on-going. Requests to access the datasets should be directed to l.hellsten@uwinnipeg.ca.

## Ethics statement

The studies involving human participants were reviewed and approved by University of Saskatchewan Behavioral Research Ethics Board. The patients/participants provided their written informed consent to participate in this study.

## Author contributions

BH is a student researcher who developed the initial draft of the manuscript under the supervision and mentorship of L-aH. L-aH is the Principle Investigator, and LM is the Co-Investigator of the related funded research project and the caretakers of the resulting dataset. All authors including BS (student researcher) contributed equally to the editing and review of the final manuscript.

## Funding

This manuscript is based on a project funded by a Social Sciences and Humanities Research Council of Canada—Insight Development Grant (430-2016-00737). The funding support allowed us to collect the data used as the foundation for this manuscript.

## Conflict of interest

The authors declare that the research was conducted in the absence of any commercial or financial relationships that could be construed as a potential conflict of interest.

## Publisher’s note

All claims expressed in this article are solely those of the authors and do not necessarily represent those of their affiliated organizations, or those of the publisher, the editors and the reviewers. Any product that may be evaluated in this article, or claim that may be made by its manufacturer, is not guaranteed or endorsed by the publisher.
